# Keratoconus1Read at the 36th annual meeting of the Medical Association of Central New York, held at Auburn, September 22, 1903.

**Published:** 1904-01

**Authors:** G. Griffin Lewis

**Affiliations:** Syracuse, N. Y.


### Keratoconus.[1]

By G. GRIFFIN LEWIS, M. D., Syracuse, N. Y.

KERATOCONUS is a peculiar pathological condition of the cornea in which it gradually becomes more or less conical in shape without any accompanying inflammation, pain, loss of transparency or other ocular symptoms, excepting a gradual failure of vision. It is much more prevalent among females and when not

1. Read at the 36th annual meeting of the Medical Association of Central New York, held at Auburn, September 22, 1903.

congenital usually begins between the tenth and fourteenth years of life, though often earlier, and proceeding very slowly, reaching its climax within from five to seven years. In any degree it may become stationary, permanently or only for awhile, and then undergo rapid increase. In some cases it may attain remarkable proportions. Dr. H. D. Noyes reported a case a few years ago which measured ¾ of an inch in length. As a rule, both eyes are affected, but one usually in advance of the other. When monocular it is generally very slight. The cone almost invariably points below the center of the cornea, which fact is probably due to the pressure of the upper lid and it never advances to bursting by extreme stretching, ulceration or sloughing.

Cases of keratoconus are comparatively rare, so much so that few are likely to come under the care of one surgeon, and it is usually well advanced when the unfortunates seek professional aid. According to the statistics of six of the largest ophthalmic hospitals in the United States there is only on an average, one case of keratoconus to every 7,206 eye cases. Literature on the subject is also very meager. The Index Medicus during the eight years from 1891 to and including 1899, only gives a total of six original articles and six reports of cases written on this subject throughout the world. The older works on ophthalmology treat more exhaustively of it than do the more recent ones. Bowman, Critchett, and Knapp perhaps have been the three most generous contributors of literature on the subject.

The causes of keratoconus may be classified into two groups, constitutional and local, the latter being dependent on the former. Heredity seems to figure somewhat in the etiology of this disease, as Bowman and others have reported several cases in the same family. Malnutrition and feeble muscular conditions brought about by severe or protracted illness, over-rapid growth, phthisis, feeble circulation, scrofula, anemia, chronic dyspepsia and menstrual disturbances have been observed to be associated with the development of conical cornea. The fact that it is more prevalent in females and that the period of puberty is the usual time of its incipiency, points very strongly to menstrual disturbances as one of the leading factors in its causation. Typhoid fever, before or at the age of puberty, may bring about this condition. Derby says, "that considerable general change may take place in the system at large as the result of typhoid fever was first brought to my notice years ago by the study of a case where an emetropic patient went to bed with an attack of typhoid fever, remained six weeks, and arose with a marked and persistent change of myopia."

The immediate local cause is the disturbance of the relation of the intraocular pressure to the resistance of the cornea, which may be due to one or more of the following conditions: (1) malnutrition, atrophy and diminished resistance power of the center of the cornea, which is farthest from the blood supply; (2) incomplete development of the center of the cornea which during fetal life is the last part to be formed; (3) muscular tension of the external ocular muscles, which in some cases has been known to exist before the conical condition of the cornea developed; (4) eye strain with the associated intraocular congestion and relative increase of tension. If the cillary muscle plays an important part on the tension and alterations of the corneal curvature and if astigmatism may, in some instances, be the direct result of such action, and that too upon the healthy cornea, why could not a severe and long continued spasm of accommodation eventually produce a conical condition of a weakened, undeveloped or badly nourished cornea?

We know nothing definite of the pathology of this malformation of the cornea, but there is no doubt but that the bulging is preceded by an atrophy of the central part of that membrane, which is farthest from its source of nutrition. Whether this atrophy of the membrane is caused by defect or deformity from birth, and yields to internal pressure in youth is still an unanswered question. Dr. His found by experimenting upon guinea pigs that when he scraped the epithelium of the cornea off, that membrane became cloudy and protruded, and that although the cloudiness cleared up after awhile the protrusion remained. Dr. J. A. Spaulding says, "In the absence of pathological alterations subsequent to keratitis I should be inclined to attribute the cause to softening of the corneal tissues from dyscrasia similar to that which gives us white swelling of the knee,—the so-called scrofulous, for lack of a better word." Ranpoldi concludes from a microscopic examination of an eye affected with keratoconus that the changes in curvature must be sought in an alteration of Descemet's membrane and its epithelium, which changes are dependent upon constitutional conditions. Bowman states that the changes are confined to the laminated tissues of the cornea, as in the specimens which he examined microscopically, the posterior laminated and the epithelium both on the front and on the back of the cone were unchanged.

When the disease is far advanced the increased friction and the exposure of the apex sets up an interstitial keratitis, but bursting never takes place. This is, no doubt, due, as Bowman says, to the exosmosis of the aqueous through the thin cornea, thus reducing the intraocular pressure so that it is no longer in excess of the diminished corneal resistance. In its earliest stages there are only subjective manifestations such as diminished acuity of vision and symptoms of asthenopia, and unless we examine very closely we will overlook the true condition. Subsequently, however, the bulging will be sufficiently advanced to give a peculiar brilliancy to the eye, like a drop of molten glass deposited upon the corneal center, and the pupil is apt to be large and sometimes the iris is tremulous. The ophthalmoscopic picture of the fundus will make the vessels appear broken and twisted. In the more advanced stages the cone may be easily seen through the closed lids as the ball is moved in different directions. The cillary vessels may become somewhat congested and the apex may grow more or less cloudy.

The accurate estimation of the refraction in these cases is very difficult on account of the distorted and wave-like sides of the cone. Vision can never be brought up to normal and in the pronounced cases glasses are of little or no use. Those cases in which vision is improved by the use of eserine or by the pinhole disc are usually benefited by glasses. It is generally assumed that the general refraction in keratoconus is myopic. This may be so in the majority of cases, but fully one-third have hyperopic astigmatism, at least in one meridian. In handling these cases we should not be governed by the precise rules which usually aid us in the correction of refractive errors, nor place too much reliance upon subjective methods, but should rely upon objective measurements. As Mackay says, "The problem before us is to estimate the actual state of the refraction through a small area of the cornea situated as near the visual axis as possible." With this object in view it is best to examine the eye first with a dilated pupil, then with a contracted pupil. The direct ophthalmoscopic method is of no assistance. Retinoscopy, however, is valuable and upon it we can depend more than on any other method.

A peculiar shadow on the side of the cone opposite the light, which circles around the cone as the mirror is moved from side to side, is characteristic of keratoconus. This shadow may confuse us somewhat in our efforts to ascertain the refractive condition, but by using a disc with an aperature of from 3 to 5 m.m. in diameter, thus screening from view every part of the cornea except the limited area which we wish to correct, the process is very much simplified. The ophthalmeter and Placido's disc are also great helps in selecting the clearest part of the cornea. Often convex cylinders are accepted. In some cases convexed cylinders placed at right angles to concave cylinders will give better vision than spherocylinders. In those cases which remain stationary stenopeic appliances sometimes render effectual assistance, but are not satisfactory for constant wear as they shut off too much light and contract the visual field. This objection may be partially overcome by having a disc pierced with a series of small holes like the cover of a pepper box. Snellen constructed a pair of discs with a stenopeic slit running from left to right and ending in the middle with a sharp point, thus enabling the patient to read when the point in the slit is brought just in the visual line. Helfrich reports a case of a young boy who devised a lens consisting of a plain glass with a horizontal black strip across the center, the width of which was sufficient to shut off those rays of light which would naturally infringe on the conical part of the cornea. With this glass his vision was improved from 5-200 to 20-50. All these appliances, however, are very much objected to on account of their unsightliness.

Rahlmann was the first one to recommend the hyperbolic lens which in most cases, improves vision remarkably as long as the visual axis corresponds to the apex of the lens, but as soon as the patient turns his eye the least to one side the condition is worse than before. For this reason they are more suitable for close work than for street wear. The same objection pertains to the conical lenses recommended by Angeluci. The contact lens suggested by Herschell, which fits on to the front of the eyeball somewhat like an artificial eye, also improves the vision very much, but irritates the eye and is not long tolerated.

The treatment of conical cornea is, as a rule, of a very unsatisfactory character and relief is only relative. Some cases have been arrested in the early stages by a general alterative treatment, absolute rest, avoidance of violent physical exertion, open air, good food, hygienic surroundings, digital massage, correction of refractive errors, compress bandages, mydriatics, myotics and astringents. Culver reports good results from the use of the thyroid gland. Surgical intervention is to be advised only after other methods have failed and the patient is reduced to a state of helplessness, for the surgical treatment of conical cornea, besides being a tedious undertaking and beset with dangers, has certain cosmetic drawbacks and doubtful gain. Sir William Bowman was the first one to attempt to correct the optical defects of conical cornea by an operation. Having noticed the improvement in vision afforded by the stenopeic slit he endeavored to supply the latter permanently by performing the operation called iridodesis, or changing the pupil into a vertical slit by drawing the papillary margin into the corneal wound and leaving it there. This operation improved vision considerably in some cases, but it occasionally provoked cyclitis and sympathetic trouble. Von Graefe had good results in some cases by shaving off the apex of the cone without entering the anterior chamber, and then applying a stick of mitigated silver caustic to the cut surface, thus producing an ulcer and eventually getting cicatricial contraction. This method was very painful and not infrequently set up a cyclitis. Bowen next invented a small trephine by which he removed a disc from the summit of the cornea. DeWecker also adopted this method and for years this was the favorite plan of treatment. Critchett then conceived the idea of removing a small elliptical piece of cornea at the apex, allowing the wound to heal without sutures. This method is also still practised by many eminent ophthalmic surgeons.

Dr. J. W. Buller described a case in 1897 in which he got an excellent result by passing a Graefe blade vertically through the apex and bandaging, then one week later passing the blade horizontally through the apex, removing a small piece of the cornea with a pair of iris scissors and bandaging again. Repeated evacuations of the aqueous humor have been employed more or less for many years, but without any pronounced result. The method most generally adopted of late years, and the one which is the least painful in its application and the least liable to subsequent complications, is cauterisation of the apex with the actual or galvanocautery. This may be applied in various ways. Abadie burns a deep furrow at the upper edge of the cornea. Callan cauterises at opposite points to the greatest curvature going down into the membrana propria of the cornea and repeats the procedure if necessary. Noyes recommended cauterisation of the cone without perforation. Weeks uses the same method. The great majority of operators, however, seem to favor Knapp's method of first cauterising the apex with an oval electrode and then piercing the eschar with a fine pointed electrode. This method is performed as follows: after dilating the pupil with atropine and cocanising the eye, an assistant gently raises the upper lid, as no speculum should be used or pressure exerted upon the eyeball. The oval electrode is placed cold over the apex and withdrawn as soon as it has been brought to a red heat. Then with a needle electrode, the size of which depends upon the amount of contraction desired, the center of the apex is pierced and the needle quickly withdrawn so as not to heat the aqueous too much and produce a traumatic cataract. For the same reason the electrodes are not allowed to reach a white heat, but are withdrawn as soon as they are brought to a red heat.

This method is easily performed, does not result in anterior synechia, allows rest and contraction of the cornea by the slow and continuous drainage of the anterior chamber and leaves a small scar. A moderately tight bandage is kept on most of the time following this operation and the increased tension which is liable to follow may be combated with eserine. A few cases of cataract, cyclitis, or even panophthalmitis have been reported to have followed this method, but thus far it has been productive of more good results and fewer bad ones than any other and may be now considered the classic treatment. In cases where a somewhat extensive eschar has been unavoidable it may subsequently be necessary to make a small artificial pupil in the line of vision, preferably inward and slightly downward. Vision and appearance will also be somewhat improved by tattooing the corneal opacity with India ink, providing that membrane is not too thin to admit of it.

600 University Block.

